# Unveiling Risankizumab’s Rare Side Effect: A Case of Severe Thrombocytopenia in Psoriatic Arthritis

**DOI:** 10.7759/cureus.81364

**Published:** 2025-03-28

**Authors:** Jinalben Chaudhari, Dighe R Prasad

**Affiliations:** 1 Internal Medicine, St. Joseph's Medical Center, Stockton, USA; 2 Internal Medicine/Hematology and Medical Oncology, St. Joseph's Medical Center, Stockton, USA

**Keywords:** il-23 inhibitors, psoriatic arthritis, risankizumab, skyrizi, thrombocytopenia

## Abstract

Drug-induced thrombocytopenia (DITP) is a rare but serious immune-mediated reaction characterized by drug-dependent antibodies that bind platelet surface glycoproteins, leading to severe thrombocytopenia. Biologic therapies, including IL-23 inhibitors like risankizumab, have been implicated in such adverse events. We present the case of a 47-year-old male with a history of psoriasis and prior deep vein thrombosis, who developed severe bleeding manifestations shortly after initiating risankizumab therapy for psoriatic arthritis. Clinical evaluation included mucocutaneous bleeding, petechiae, and a precipitous drop in platelet count to 3 x 10^3/uL. Management strategies involved platelet transfusions, high-dose steroids, intravenous immunoglobulin (IVIG), and thrombopoietin receptor agonists due to inadequate initial response. Despite aggressive treatment, the patient's thrombocytopenia persisted, necessitating prolonged hospitalization and consideration of alternative therapies. This case underscores the critical importance of recognizing and managing rare hematologic complications associated with biologic therapies. Vigilance in monitoring platelet counts during IL-23 inhibitor therapy is essential to mitigate severe adverse outcomes.

## Introduction

Drug-induced thrombocytopenia (DITP) manifests as an idiosyncratic immune-mediated reaction mediated by drug-dependent antibodies that selectively bind platelet surface glycoproteins in the presence of specific drugs [1,2]. These antibodies gain affinity for platelet epitopes upon exposure to sensitizing medications, typically within one to two weeks of initiation. Notably, drugs with charged or hydrophobic structural elements facilitate binding to both antibodies and platelet proteins, triggering thrombocytopenia only when the drug is present [3]. Withdrawal of the offending drug usually leads to rapid resolution of thrombocytopenia, underscoring its drug-dependent nature. Despite the challenges in diagnosing DITP due to limited reporting, varied assay sensitivities, and potential metabolite involvement, it remains crucial to consider in patients presenting with unexplained thrombocytopenia, especially those experiencing recurrent episodes following drug exposure [4,5]. DITP symptoms can be very similar to those of other conditions, such as idiopathic thrombocytopenic purpura (ITP), viral infections, or hematologic disorders. The presence of petechiae, bruising, or bleeding might not immediately raise suspicion of DITP without further clinical evaluation. The onset of thrombocytopenia due to DITP often occurs several days after the drug has been administered. This delay in time complicates the diagnosis, as healthcare providers may not immediately consider a recent medication as a potential cause of the platelet drop. DITP is a rare condition, and many healthcare providers may not be aware of it or might not consider it in the differential diagnosis. This can lead to missed diagnoses, as the condition requires a high index of suspicion and familiarity with drug-induced reactions.

Here we present a case of a 47-year-old male presenting with significant bruising, petechiae, and mucosal bleeding after six weeks of initiating risankizumab. This case report illustrates a rare but significant adverse event associated with risankizumab therapy. Clinicians should maintain vigilance for unusual hematologic manifestations, especially in patients with predisposing factors such as prior thrombotic events or unexplained bleeding. Further research and reporting of such cases are crucial to better understand the full spectrum of adverse effects associated with risankizumab and similar IL-23 inhibitors [6,7]. IL-23 is a pro-inflammatory cytokine that plays a central role in the activation and differentiation of T-helper 17 (Th17) cells, which are crucial in the pathogenesis of various autoimmune diseases. IL-23 stimulates the production of other pro-inflammatory cytokines such as IL-17A, IL-17F, and IL-22, which further contribute to inflammation, tissue damage, and the development of autoimmune diseases. By inhibiting IL-23, these drugs aim to reduce the activity of Th17 cells, subsequently decreasing inflammation and improving symptoms in diseases like psoriasis and inflammatory bowel disease (IBD).

## Case presentation

A 47-year-old male with a past medical history significant for psoriasis and a history of deep vein thrombosis (DVT) presented to the emergency department with sudden-onset gum bleeding persisting for three days, prompting him to discontinue apixaban. The patient's clinical presentation was marked by large erythematous scaly patches consistent with psoriasis on his back and bilateral legs. Examination also revealed scattered petechiae across his abdomen and arms and superficial ulcers on his lower lip. Although the lip ulcers were not actively bleeding, they indicated a potential for mucocutaneous involvement. The patient reported generalized musculoskeletal pain and specific complaints of back pain, consistent with his history of psoriatic arthritis. 

Patient has been managing psoriatic arthritis with prior treatments including Humira and Cosentyx. However, risankizumab, an IL-23 inhibitor, was chosen due to its distinct mechanism of action, which targets the IL-23/IL-17 pathway, offering potential benefits in this patient because he had not responded optimally to tumor necrosis factor (TNF) inhibitors (like Humira) or IL-17 inhibitors (like Cosentyx). Risankizumab, a newer therapy, was initiated six weeks before this presentation. Additionally, he has a history of extensive right leg DVT necessitating thrombectomy and has been managed with anticoagulation therapy. The presence of a previous thrombotic event may suggest an increased risk for thrombosis or clotting abnormalities, which could be relevant in evaluating the patient’s platelet count and any potential drug-related interactions that could exacerbate or complicate their condition. Despite this, he had been off anticoagulation recently, as deemed appropriate based on prior evaluation for thrombophilia, which yielded negative results. Reevaluation for potential future anticoagulation had been recommended by hematology/oncologist due to the elapsed time since his DVT. Laboratory assessments were significant for a white blood cell count of 10.5 x 10^3/uL, a red cell distribution width of 15%, platelets at 3 x 10^3/uL, mean platelet volume of 10.7 fL, prothrombin time of 12.0 seconds, and partial thromboplastin time of 36.9 seconds, as seen in Table 1. 

**Table 1:** The patient's significant laboratory findings upon admission.

Parameter	Normal Range	Result
White Blood Cells (WBC)	4.0 - 11.0 x 10^3/uL	10.5 x 10^3/uL
Red Cell Distribution Width (RDW)	11.5% - 14.5%	15%
Platelets	150 - 450 x 10^3/uL	3 x 10^3/uL
Mean Platelet Volume (MPV)	7.4 - 10.4 fL	10.7 fL
Prothrombin Time (PT)	10.0 - 13.0 seconds	12.0 seconds
Partial Thromboplastin Time (PTT)	25.0 - 36.0 seconds	36.9 seconds

In the Emergency Department, he received a platelet transfusion and IV 1000 mg of methylprednisolone. Following admission to the med-surg unit, Hematology/Oncology was consulted due to ongoing bleeding and concerns regarding his thrombotic risk. 

Upon admission, he was started on IV dexamethasone 40 mg daily, IVIG, and IV Protonix 40 mg twice a day. Eliquis was held due to the bleeding episode, and a duplex venous ultrasound revealed a nonocclusive DVT in the right popliteal vein. Hematology recommended close monitoring of the DVT and initiated high-dose steroids alongside IVIG to manage his thrombocytopenia. 

Over the subsequent days, despite these interventions, his platelet count remained low at 16K, prompting consideration of romiplostim (Nplate). A bone marrow biopsy was performed to further investigate the etiology of his thrombocytopenia, which showed unremarkable bone marrow with normocellular features and some megakaryocytic and myeloid hyperplasia. 

Continued management included ongoing high-dose steroid therapy, IVIG, and the introduction of Nplate. Complications arose from steroid use, notably steroid-induced hyperglycemia, necessitating initiation of an insulin regimen. Daily platelet counts were monitored, and liver function tests were ordered to assess treatment response and potential side effects. 

By day eight to 10 of hospitalization, his platelet count showed minimal improvement despite Nplate therapy, prompting consideration of alternative treatments such as fostamatinib, with plans for rituximab if his condition failed to improve. Further investigations were conducted to assess potential rheumatologic conditions that could be contributing to the patient's thrombocytopenia. Autoimmune marker testing revealed antinuclear antibody (ANA) titers of 1:80 (normal range: <1:40), which can suggest an autoimmune process, although low titers can also be seen in healthy individuals. The erythrocyte sedimentation rate (ESR) was markedly elevated at 86 mm/hr (normal range: <20 mm/hr for women), indicating significant systemic inflammation. Additionally, the C-reactive protein (CRP) level was 1.45 mg/dL (normal range: <1.0 mg/dL), further supporting the presence of ongoing inflammatory activity. These results, in conjunction with the clinical presentation, were used to explore possible underlying autoimmune or inflammatory causes of the thrombocytopenia.

**Figure 1:**
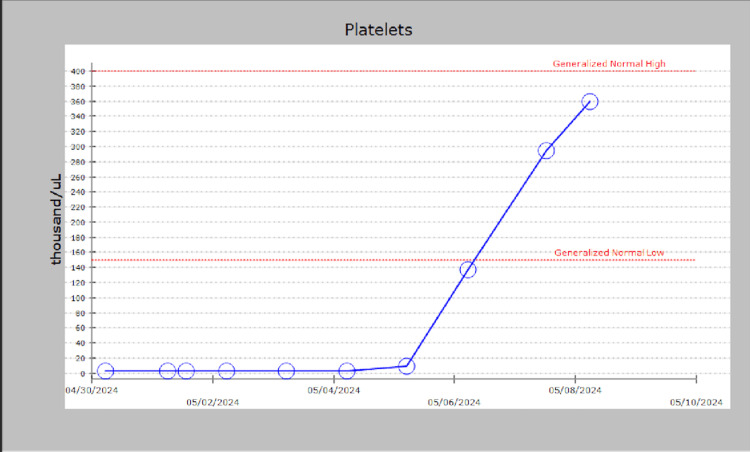
The x-axis represents the duration of hospitalization, measured in days, while the y-axis indicates the platelet count (in thousands per microliter). This graph illustrates the time it took for the platelet count to normalize throughout the patient's hospital course.

On days 11 to 13, after withdrawal from the offending medication (risankizumab), there was notable improvement as his platelet count increased to 137 K by day 12. This improvement allowed for initiation of prednisone tapering and consideration of fostamatinib. Additional investigations revealed a small IgG kappa monoclonal protein spike on serum protein electrophoresis. With stable clinical status and normalized platelet counts (360 K on day 13) as seen in Figure 1, the patient was discharged with instructions for monitoring the monoclonal protein and plans for outpatient follow-up. 

Upon discharge, instructions included scheduling repeat tests serum protein electrophoresis (SPEP), serum immunofixation electrophoresis (IFE), serum free kappa and lambda chains in six months. The patient was advised to continue Tavalisse according to discharge instructions and educated on recognizing signs of bleeding and when to seek medical attention. Plans for potential interventions such as splenectomy were discussed if initial therapies were unsuccessful, emphasizing ongoing monitoring for DVT and adjustment of anticoagulation as recommended by the hematologist. 

## Discussion

Drug-induced thrombocytopenia presents a diagnostic challenge due to its potential causation by numerous medications, often leading to oversight amidst the broad differential diagnosis of thrombocytopenia [1,2]. This oversight is particularly notable in outpatient settings, where patients may be misdiagnosed and treated for autoimmune thrombocytopenia before the offending drug is identified [3]. Similarly, in hospitalized patients, thrombocytopenia is frequently attributed to underlying conditions, further complicating the recognition of DITP [4].

Idiosyncratic drug-sensitivity reactions like DITP affect only a minority of individuals exposed to potential culprit medications, with no discernible genetic or environmental predispositions identified thus far [5]. Understanding the mechanisms underlying immune-mediated thrombocytopenia induced by drugs is crucial for accurate diagnosis and timely management of DITP in clinical practice [6].

The evaluation of DITP requires meticulous clinical assessment and exclusion of alternative causes of thrombocytopenia. It is important to note the temporal association between drug administration and thrombocytopenia onset, sustained recovery post-drug cessation, exclusion of other etiologies, and recurrence upon re-exposure to the suspected medication [7]. Therefore, while confirmation via drug-dependent anti-platelet antibody testing is ideal, clinical judgment and prompt cessation of suspected drugs remain pivotal in managing DITP [8].

Drug-induced thrombocytopenia typically presents with a sudden and severe decrease in platelet counts, often dropping below 20,000/μL, which can lead to significant bleeding complications and, in rare cases, fatalities [1]. Prompt recognition and discontinuation of the offending drug are critical, as thrombocytopenia usually begins to resolve within days after cessation, and complete recovery is expected within a week [2]. Management may necessitate platelet transfusions to manage bleeding episodes, and corticosteroids are often administered empirically due to the initial difficulty in distinguishing DITP from immune thrombocytopenia [5]. Thrombocytopenia can result from various mechanisms, including decreased platelet production due to bone marrow disorders like aplastic anemia, myelodysplastic syndromes, or leukemia, as well as nutritional deficiencies (e.g., vitamin B12 or folate), and infections like HIV or parvovirus B19. Increased platelet destruction is commonly seen in conditions like ITP, drug-induced thrombocytopenia (e.g., heparin, quinine, IL-23 inhibitors), systemic lupus erythematosus (SLE), and HIV. Thrombotic microangiopathies, including hemolytic uremic syndrome (HUS) and thrombotic thrombocytopenic purpura (TTP), and disseminated intravascular coagulation (DIC). 

IL-23 inhibitors like risankizumab, ustekinumab, and guselkumab are pivotal in managing conditions such as psoriatic arthritis and psoriasis. While effective, these biologics have been associated with rare occurrences of thrombocytopenia, as highlighted in case reports [3,6]. Similarly, our case presents with severe thrombocytopenia following the initiation of risankizumab, another IL-23 inhibitor, underscoring the need for vigilance in monitoring platelet counts during treatment [4].

These observations emphasize the importance of recognizing and managing potential hematologic adverse effects in patients undergoing biologic therapy for chronic inflammatory conditions. Given that the patient has psoriasis, this is an important factor to consider as it increases the likelihood of immune system dysregulation, which could contribute to DITP. Psoriasis itself is an autoimmune condition, and patients with psoriasis are often treated with biologics, such as IL-23 inhibitors (e.g., risankizumab), which are effective in managing the inflammation associated with the disease. However, biologic medications, especially IL-23 inhibitors, have been associated with rare cases of thrombocytopenia due to immune-mediated platelet destruction.

In this case, the patient's psoriasis may predispose them to immune-mediated reactions, making them more susceptible to developing DITP, particularly with the initiation of a new biologic therapy like risankizumab. Additionally, if the patient has used other biologics such as Humira (adalimumab) or Cosentyx (secukinumab) in the past, this might also suggest a history of immune modulation that could increase the risk of drug-induced adverse reactions.

Thus, while psoriasis itself isn't a direct cause of DITP, the immune system dysregulation seen in autoimmune diseases like psoriasis and the use of biologic therapies are relevant predisposing factors that should be considered when assessing the potential link to thrombocytopenia in this patient. 

Risankizumab, a humanized monoclonal antibody targeting interleukin-23, is efficacious in treating moderate to severe plaque psoriasis and psoriatic arthritis but carries potential risks of uncommon adverse effects, including drug-induced thrombocytopenia. This case underscores the critical need for vigilance in recognizing and managing such adverse reactions.

Management of risankizumab-induced thrombocytopenia required a multidisciplinary approach, including high-dose steroids, and IVIG. Fostamatinib and romiplostim (Nplate) were specifically indicated for this patient because he did not respond to first-line therapies like corticosteroids or intravenous immunoglobulin to stabilize platelet count. Discontinuation of risankizumab was crucial, followed by careful monitoring and management of thrombocytopenia. As risankizumab is a relatively new IL-23 inhibitor, its safety profile primarily derives from clinical trials and post-marketing surveillance, highlighting the importance of ongoing awareness among clinicians regarding less frequently reported adverse events [7,8].

## Conclusions

Drug-induced thrombocytopenia associated with risankizumab has not been widely reported in the literature, and our understanding is primarily based on isolated case reports and limited clinical data. As a result, it is important to maintain vigilance and continue reporting adverse events to further assess the safety profile of risankizumab and other emerging therapies for immune-mediated inflammatory diseases. While risankizumab is generally well-tolerated, like other biologic agents, it may rarely cause bone marrow suppression or other hematologic side effects, including thrombocytopenia. Furthermore, the inflammatory nature of psoriatic arthritis itself can contribute to systemic inflammation, potentially influencing platelet counts. 

This discussion integrates clinical findings with current literature to emphasize the importance of recognizing and managing rare adverse reactions associated with novel biologic therapies like risankizumab. Given the growing use of IL-23 inhibitors in treating autoimmune diseases, further research is needed to fully understand their safety profile, including their potential hematologic adverse effects like thrombocytopenia. Larger, well-designed studies focusing on the long-term safety and risks of risankizumab and other IL-23 inhibitors could provide clinicians with more data to make informed decisions about their use, particularly in patients with complex medical histories.
